# Necrological

**Published:** 1881-07

**Authors:** 


### NECROLOGICAL.

"Death is the crown of life:
Were death denied, poor man would live in vain."

Dr. J. William Walls, of Baltimore, died very suddenly in Philadelphia, on the 19th of May ultimo. Dr. Walls was a native of Harper's Ferry, Virginia, and was a graduate of the Winchester Medical College, a school founded by the late Dr. Hugh Maguire, of that town. Dr. Walls was Professor of Anatomy and Physiology in his *alma mater*, at the breaking out of the civil war. He then joined the Confederate army and served with ability, as a surgeon, until the close of the war, since which he has been engaged in practice in Baltimore.

